# Screening and Evaluation of Quinoa Germplasm for Saline–Alkali Tolerance Based on Germination Vigor Traits

**DOI:** 10.3390/plants15152413

**Published:** 2026-08-06

**Authors:** Qinghan Bao, Xiaowei Wei, Yang Wu, Yongping Zhang, Yue Zhang, Yang Wang

**Affiliations:** 1 Jilin Provincial Key Laboratory of Plant Resources Science and Green Production, Jilin Normal University, Siping 136000, China; 2 College of Agriculture, Inner Mongolia Agricultural University, Hohhot 010018, China

**Keywords:** quinoa, germination, compound saline–alkali stress, multivariate analysis, tolerance evaluation

## Abstract

Soil salinization and alkalization caused by global climate change have become major constraints on crop production worldwide. Quinoa (*Chenopodium quinoa* Willd.), owing to its exceptional adaptability to adverse environmental conditions, is considered a promising crop for saline–alkali agriculture. Successful screening and evaluation of saline–alkali-tolerant germplasm at the germination stage is essential for breeding quinoa varieties with enhanced tolerance to compound saline–alkali stress. In this study, 23 quinoa accessions originating from different regions were evaluated for saline–alkali tolerance by exposing seeds to compound saline–alkali solutions at different concentrations in a Petri dish germination assay. During the germination stage, relative vigor index, germination rate, germination energy, shoot length, root length, and fresh weight were determined, and saline–alkali tolerance was assessed using multivariate statistical analyses. The germination ability and seedling growth potential of quinoa germplasm gradually declined with increasing saline–alkali stress. Based on the comprehensive saline–alkali tolerance index at the germination stage, eight accessions were identified as highly tolerant and four accessions as highly sensitive to saline–alkali stress. Among them, LL1 and Z27 exhibited outstanding tolerance and were identified as elite saline–alkali-tolerant germplasm resources. The results indicated that vigor index and fresh weight at the germination stage can serve as key parameters for evaluating saline–alkali tolerance in quinoa germplasm. Overall, this study provides valuable germplasm resources and a practical basis for breeding new quinoa varieties with enhanced saline–alkali tolerance, as well as for the identification and utilization of stress-resistance-related genes in quinoa.

## 1. Introduction

Soil salinization and alkalization represent one of the most severe abiotic threats to global agricultural sustainability [1], currently affecting over 1 billion hectares of land worldwide. This widespread stressor triggers a cascade of detrimental edaphic and physiological consequences, including severe soil compaction [2], nutrient imbalance in root zones [3], and persistent physiological drought in crop plants [4]. In China alone, approximately 7.6 million hectares of arable and marginal land are classified as saline-affected, with the Songnen Plain in Jilin Province representing one of the most ecologically and agronomically critical regions dominated by soda saline–alkali soil, where NaHCO_3_ and Na_2_CO_3_ are the primary phytotoxic salt components. Compound saline–alkali stress induces complex phytotoxic effects, including ionic toxicity [5], osmotic stress [6], high pH [7], and oxidative damage [8,9], which collectively inhibit seed germination, stunt seedling growth, and significantly reduce crop yield [10]. Developing and cultivating stress-tolerant crops is considered a sustainable strategy to improve saline land use efficiency and ensure agricultural productivity.

Quinoa (*Chenopodium quinoa* Willd.), a highly nutritious pseudocereal, has garnered increasing global attention due to its exceptional and well-documented nutritional profile [11,12], which features a uniquely balanced composition of essential amino acids, high-quality proteins, dietary fibers, vitamins, and health-promoting minerals. This outstanding nutritional composition has positioned quinoa as a strategically important crop for advancing global food security and diversified human nutrition [13,14]. Notably, quinoa also exhibits remarkable natural resilience to a broad spectrum of abiotic stresses, including salinity [15,16,17], drought [18,19,20], and frost [21,22,23], enabling it to complete its life cycle and produce harvestable yields in harsh marginal environments where most conventional cereal crops fail. Classified as a facultative halophyte, quinoa has been reported to maintain stable growth at NaCl concentrations ranging from 75 to 150 mmol/L [24,25], making it an exceptionally promising candidate for both the agronomic utilization and phytoremediation of widespread saline–alkaline soils [26]. However, accurately evaluating crop tolerance to this type of multifaceted compound stress remains a major technical challenge that cannot be reliably addressed by traditional single-parameter assessment methods and instead requires systematic, multi-indicator integrated evaluation frameworks.

Previous studies have generated valuable preliminary insights into quinoa’s physiological responses to singular NaCl salt stress [27,28,29] or isolated alkali stress induced by NaHCO_3_ or Na_2_CO_3_ during the germination stage [30,31,32]. Nevertheless, these simplified experimental conditions do not accurately replicate the complex, field-realistic properties of natural saline–alkaline soils, where multiple coexisting ions (Na^+^, Cl^−^, SO_4_^2−^, HCO_3_^−^, CO_3_^2−^) exert combined, synergistic phytotoxic effects that cannot be fully captured by single-salt or single-alkali treatments. As a direct consequence, the tolerance mechanisms characterized and tolerant germplasm identified under these simplified singular stress conditions often fail to maintain their expected high performance when transplanted to actual compound saline–alkali field environments. Critically, the seed germination and early seedling establishment stage is widely recognized as the most sensitive developmental window to abiotic stress, and it serves as a highly efficient, time-saving, and reliable phase for large-scale preliminary screening of stress-tolerant germplasm resources [33,34]. To date, however, systematic research focusing on quinoa’s germination and post-germination tolerance to ecologically relevant compound saline–alkali stress remains notably scarce.

In recent years, the evaluation of crop stress tolerance has undergone substantial methodological advancement, evolving from overreliance on single or isolated physiological indicators toward integrated analytical frameworks that deliver more objective, robust, and reproducible phenotypic assessments under adverse conditions [35,36,37]. Multivariate statistical approaches, including principal component analysis and fuzzy membership function evaluation, have been widely validated as powerful, standardized tools for quantifying comprehensive stress tolerance across diverse crop species [38,39]. Against this background, the present study was designed to systematically screen and characterize saline–alkali-tolerant germplasm accessions from a panel of quinoa cultivars cultivated across distinct ecological regions. The research procedure is as follows: based on key morphological indicators during the germination stage, superior saline–alkali-tolerant germplasms were preliminarily selected from 23 quinoa varieties tested. These 23 varieties were then further evaluated for comprehensive saline–alkali tolerance at the post-germination stage, leading to additional screening of highly tolerant materials. The overarching objective of this work is to identify a core set of highly saline–alkali-tolerant quinoa germplasms and establish a robust, practicable set of evaluation indicators that can support efficient and reliable screening for saline–alkali tolerance in quinoa breeding programs. This study is expected to provide valuable genetic resources and a methodological reference for advancing stress-adaptation research in quinoa while supporting the sustainable utilization of saline–alkali marginal lands.

## 2. Materials and Methods

### 2.1. Plant Materials and Growth Conditions

A total of 23 quinoa germplasm accessions were used in this study. Among them, LL1 was provided by the Gansu Academy of Agricultural Sciences, and the remaining materials were supplied by the Institute of Crop Sciences, Chinese Academy of Agricultural Sciences. Detailed information, including origin and thousand-grain weight (TGW), is presented in Table 1. Seeds were stored at 4 °C in a dry environment before use.

### 2.2. Stress Treatment and Experimental Design

Two treatments were established for control (CK, distilled water) and compound saline–alkali stress (SA) [40,41,42]. The SA solution was prepared by mixing NaCl, Na_2_SO_4_, NaHCO_3_, and Na_2_CO_3_ at a molar ratio of 1:1:1:1. The stated concentrations refer to the total concentration of all four salts combined, with each individual salt contributing equally at a 1:1:1:1 molar ratio. For example, in the 50 mmol/L treatment, each salt is present at 12.5 mmol/L.

Four concentration gradients (50, 100, 200, and 300 mmol/L) were initially tested using five accessions (A4, Z27, LL1, D2, 16ND-3) randomly selected from diverse geographical origins to ensure broad representation. The optimal concentration was defined as the lowest level that caused substantial inhibition of seedling growth while maintaining germination rate above 90% of the control, a threshold that ensures sufficient biological replicates for subsequent trait evaluation and maximizes the discriminatory power among accessions.

The pH of the freshly prepared SA solutions was measured using a calibrated pH meter (PHS-25, Shanghai Inesa Science Instrument Co., Ltd., Shanghai, China) at (25 ± 1) °C. Based on preliminary results (see Section 3.1), 50 mmol/L was selected as the optimal concentration for germplasm screening, as it imposed moderate inhibition (germination rate ~0.93 of the control) without complete suppression.

Uniform, plump quinoa seeds were surface-sterilized with 10% sodium hypochlorite for 15 min, rinsed three times with deionized water, and dried on sterilized filter paper. Thirty seeds were evenly placed in Petri dishes (90 mm diameter) lined with two layers of filter paper, with three replicates per treatment. Each Petri dish (containing 30 seeds) was considered one experimental unit (biological replicate), with three replicate dishes per treatment. Control dishes received 10 mL of distilled water; stress dishes received 10 mL of 50 mmol/L SA solution. All dishes were placed in an artificial climate incubator set at (25 ± 1) °C, relative humidity (60 ± 5)%, with a 12 h/12 h light/dark photoperiod and light intensity of 200 µmol·m^−2^·s^−1^. Filter papers were replaced daily, and an appropriate amount of distilled water or SA solution was replenished to maintain a constant culture environment.

### 2.3. Measurement of Germination and Growth Traits

Seed germination (indicated by radicle emergence) was recorded every 24 h. The following indices were calculated as follows:

Germination energy (GE) = (G_4_/total number of seeds) × 100%

Germination rate (GR) = (G_8_/total number of seeds) × 100%

Germination index (GI) = Σ (G_t_/D_t_)

Vigor index (VI) = GI × BL

Definitions of symbols are listed below:

G_4_: number of germinated seeds on the 4th day;
G_8_: number of germinated seeds on the 8th day;
G_t_: number of germinated seeds on day t;
D_t_: the corresponding counting day.

On day 8, three seedlings were randomly picked from each replicate plate to determine root length (RL), bud length (BL), and fresh weight (FW). The mean value of the three sampled seedlings was taken as the datum of one replicate, and the resulting three replicate datasets were subjected to subsequent statistical analysis.

### 2.4. Statistical Analysis and Evaluation Methods

The relative value of each indicator under stress conditions was calculated as follows [43]:

Relative value = (mean value under SA)/(mean value under CK)(1)

Suitability of PCA was confirmed by the Kaiser–Meyer–Olkin (KMO) measure (0.732) and Bartlett’s test of sphericity (*p* < 0.001). Components with eigenvalue > 1 and cumulative contribution rate > 80% were retained.

Membership function value for each comprehensive indicator was calculated as follows:

*μ*(*X_j_*) = (*X_j_* − *X_min_*)/(*X_max_* − *X_min_*) (2)

where *μ*(*X_j_*) is the membership value of the *j*-th indicator; *X_j_* is the value of that indicator, and *X_min_* and *X_max_* are the minimum and maximum values of that indicator across all accessions.

The weight of each comprehensive indicator was

***CI****_j_* = *V_j_*/∑ *V_j_* (3)

where *CI_j_* is the weight of the *j*-th comprehensive indicator; *V_j_* is the contribution rate (%) of the *j*-th principal component.

The comprehensive evaluation value (*D*) for each accession was

*D* = ∑ [*μ*(*X_j_*) × ***CI****_j_*] (4)

### 2.5. Cluster Analysis and Stepwise Regression

Hierarchical cluster analysis was performed on the *D* values using between-groups linkage and squared Euclidean distance (SPSS 22.0). Stepwise regression was conducted with the D value as the dependent variable and the seven relative trait values as independent variables (entry criterion: F-to-enter ≥ 3.84; removal: F-to-remove ≤ 2.71).

### 2.6. Statistical Software and Reproducibility

Data were processed using Excel 2019. All experiments included three biological replicates, and data are presented as means ± standard deviation (SD). Statistical analyses were performed using SPSS 22.0. Normality was checked using the Shapiro–Wilk test, and homogeneity of variances was assessed by Levene’s test. For multiple comparisons among treatments, a one-way analysis of variance (ANOVA) followed by Duncan’s multiple-range test was applied at a significance level of α = 0.05. For percentage traits, including GR, GE, GI, VI, RL, BL, and FW, data were arcsine-square-root-transformed prior to ANOVA to satisfy normality assumptions. Pearson correlation coefficients were calculated for correlation analysis, as the data met normality assumptions. Boxplots were generated using OriginPro 2025.

## 3. Results

### 3.1. Screening for Optimal Compound Saline–Alkali Stress Level

To determine an appropriate stress concentration that effectively differentiates tolerance levels among quinoa accessions, five accessions (A4, Z27, LL1, D2, 16ND-3) randomly selected from diverse geographical origins were subjected to SA solutions at 50, 100, 200, and 300 mmol/L. The pH of the freshly prepared solutions was 10.12, 10.05, 9.94, and 9.84, respectively (Table S1). A gradual pH decrease with increasing concentration was observed, likely due to the ionic strength effect reducing CO_3_^2−^ activity.

**Figure 1:**
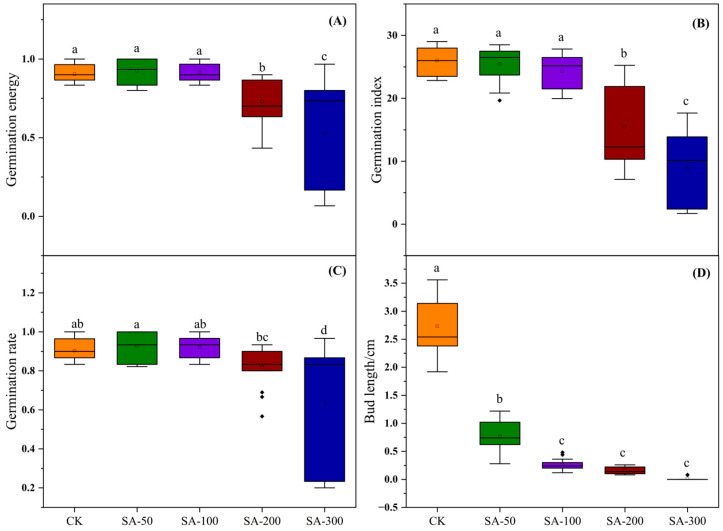
The effect of different stress concentrations on germination indicators of quinoa germplasm: (**A**) germination energy; (**B**) germination index; (**C**) germination rate; (**D**) bud length. Different letters mean a significant difference at the 5% level.

A critical consideration in germplasm screening is the selection of an appropriate stress concentration. An ideal screening level should satisfy two often-contradictory requirements: imposing sufficient stress to reveal genetic diversity, while preserving enough viable germinated seeds to allow accurate measurement of growth parameters across all entries. As shown in Figure 1 and Table S1, the 200 and 300 mmol/L treatments caused severe inhibition of GR, GE, GI, and BL compared with 50 mmol/L (*p* < 0.05), with the 300 mmol/L treatment resulting in near-complete suppression of seedling growth, thereby precluding meaningful evaluation of post-germination traits for most accessions. Compared with the 50 mmol/L treatment, the 100 mmol/L treatment showed a significant reduction in BL (*p* < 0.05), while no statistically significant differences were observed in GR, GE, and GI between the two concentrations. In addition, no significant differences in BL were detected among the 100, 200, and 300 mmol/L treatments, and all three high-concentration treatments exerted destructive effects on BL. Therefore, 100 mmol/L is not suitable as an optimal concentration for stress screening. The 50 mmol/L mixed solution caused severe yet non-lethal inhibition: BL only retained 28.1% of the control level. Nevertheless, the germination rate stayed higher than 92% relative to the control group. Each replicate retained at least 27 viable seedlings, which supplied sufficient samples to measure root length and fresh weight for all 23 germplasm materials. Therefore, 50 mmol/L was selected as the optimal stress level for germplasm screening: it strikes a balance between maintaining biological viability for trait evaluation and maximizing the phenotypic resolution necessary for distinguishing tolerant from sensitive genotypes. This concentration-setting principle is particularly relevant for compound saline–alkali stress studies, where high pH and ion toxicity impose constraints that are concurrent yet mechanistically distinct, affecting germination and early seedling growth differently.

TGW of the 23 accessions ranged from 1.238 g (F2) to 2.417 g (LL1) (Table 1). No significant correlation was found between TGW and any of the relative stress-tolerance indices, indicating that seed size per se does not predict compound saline–alkali tolerance during germination.

### 3.2. Effects of Compound Saline–Alkali Stress on Germination Traits and Correlation Analysis

The relative values of seven indicators for 23 accessions under 50 mmol/L stress are presented in Table 2. The coefficient of variation (CV) ranged from 0.147 (GR) to 0.460 (VI), revealing that the vigor index was the most stress-sensitive parameter, whereas germination rate was the least responsive. Notably, the maximum relative GR exceeded 1.0 (1.071 for K2), suggesting a possible hormetic effect of low mixed-salt concentrations in certain accessions. In marked contrast, the mean relative values of RL and VI were extremely low (0.088 and 0.084, respectively), underscoring how seedling growth parameters are far more constrained than germination initiation under compound stress.

**Figure 2:**
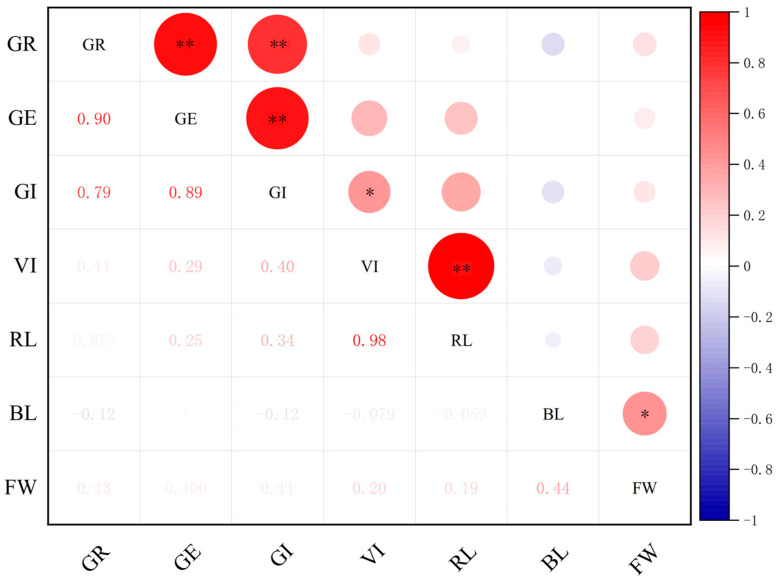
Correlation among the relative value of compound saline–alkali stress indexes of quinoa germplasm. The color depth, circle size and the transparency of the correlation coefficient values are positively associated with the absolute value of the correlation coefficients: darker colors, larger circles and clearer numbers indicate stronger correlations, while lighter colors, smaller circles and faded values indicate weaker correlations. Blue and red circles represent positive and negative correlations, respectively. * and ** indicate significant correlation at 0.05 and 0.01 probability level, respectively.

Correlation analysis (Figure 2) revealed significant positive correlations among GR, GE, and GI (*r* = 0.789 ~ 0.900, *p* < 0.01). VI was significantly correlated with RL (*r* = 0.982, *p* < 0.01) and GI (*r* = 0.402, *p* < 0.05). No strong negative correlations were observed among any indicators, indicating that no single trait trades off against another under this stress regime. These strong interdependencies justify the use of PCA to avoid multicollinearity.

### 3.3. Principal Component Analysis of Saline–Alkali Tolerance Indicators

**Figure 3:**
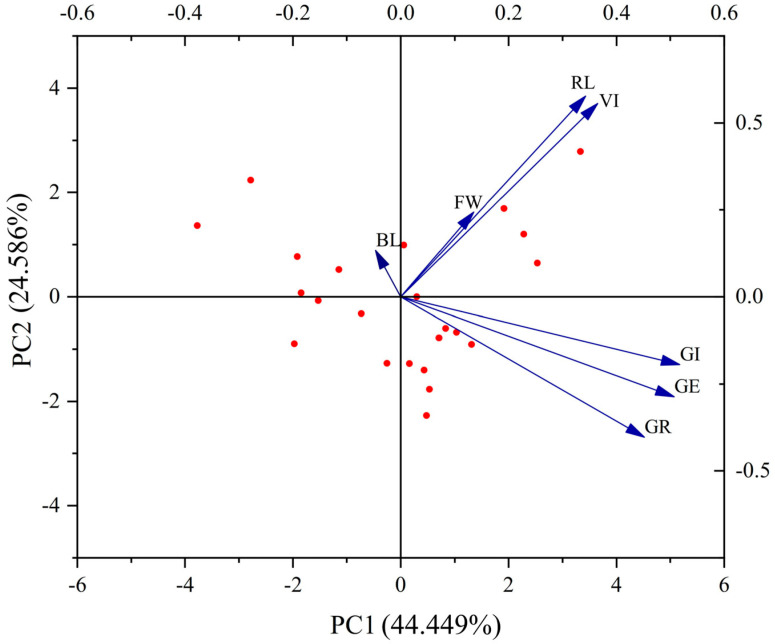
Principal component analysis of the compound saline–alkali stress index for each morphological parameter at the germination stages of 23 quinoa varieties. Red vectors represent trait loadings, with vector length indicating contribution strength and vector angles reflecting trait correlations.

To transform the seven correlated indicators into a smaller set of independent comprehensive indices, PCA was performed (Figure 3, Table 3). Based on the criterion of eigenvalue > 1 and cumulative contribution rate > 80%, the first three principal components (PCs) were retained. PC I, PC II, and PC III had eigenvalues of 3.111, 1.721, and 1.412, explaining 44.449%, 24.587%, and 20.167% of the total variance, respectively, with a cumulative contribution of 89.203%.

The eigenvector matrix (component coefficient matrix) (Figure 3, Table 3) shows that PC I was primarily loaded by GR, GE, and GI (coefficients 0.287, 0.256, and 0.293, respectively), collectively representing the overall seed germination capacity. Among these three traits, GE (germination energy, recorded on day 4) primarily reflects the speed and uniformity of germination initiation, GR (germination rate, recorded on day 8) represents the final germination ability, and GI (germination index) integrates both speed and cumulative germination throughout the germination period. Thus, PC I captures a composite dimension of germination performance that encompasses initiation speed, progression uniformity, and final success rather than any single aspect alone. PC II was heavily loaded by VI and RL (0.423, 0.440), capturing seedling vigor and root development. PC III was dominated by BL and FW (0.606, 0.537), reflecting aboveground biomass accumulation. This factorial structure reveals that compound saline–alkali tolerance in quinoa is a multidimensional trait involving distinct physiological processes.

### 3.4. Comprehensive Evaluation of Saline–Alkali Tolerance Using D Value

Based on the membership function values derived from the three PCs and their weights (49.829%, 27.563%, 22.608%; calculated from contribution rates), a comprehensive *D* value was computed for each accession (Table 4). LL1 exhibited the highest *D* value (0.806), followed by Z27 (0.786) and 16ND-3 (0.723), indicating superior overall tolerance. In contrast, D2 (0.302), D3 (0.293), and H4 (0.262) ranked lowest, suggesting high sensitivity. Notably, an accession with strong tolerance in one PC did not necessarily rank high in others (e.g., LL1 scored highest in PC I and PC II but relatively low in PC III), underscoring the necessity of a comprehensive index that integrates all stress-response dimensions.

### 3.5. Cluster Analysis Validates the Classification of Tolerance Groups

**Figure 4:**
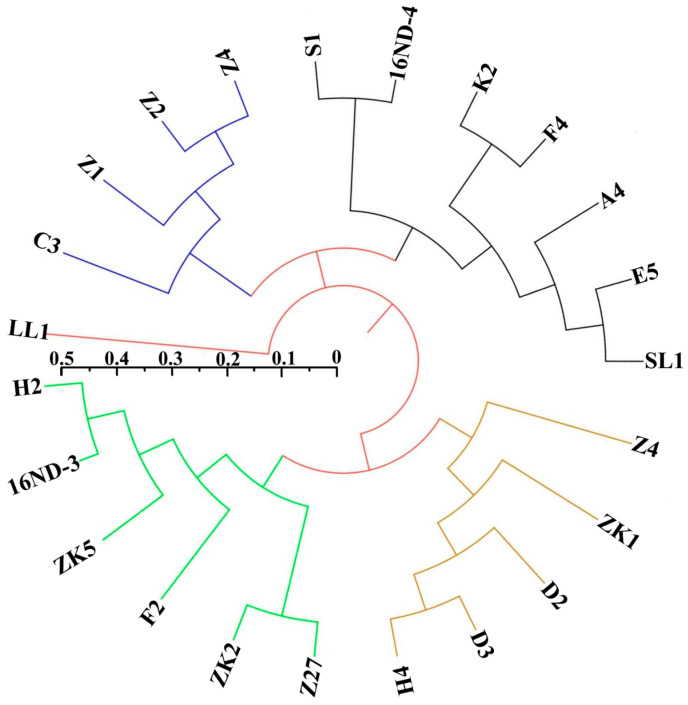
Cluster dendrogram of compound saline–alkali tolerance of 23 quinoa varieties.

Using the *D* values, hierarchical cluster analysis (Figure 4) classified the 23 accessions into three distinct groups at a Euclidean distance of 7:

Group I (strong tolerance): eight accessions (LL1, Z27, 16ND-3, H2, ZK5, ZK2, C3, F2), accounting for 34.78% of the tested materials. These accessions consistently showed above-average *D* values and can be prioritized for breeding.

Group II (moderate tolerance): eleven accessions (47.83%), including Z1, Z2, 16ND-4, S1, K2, F1, E5, A4, SL1, Z4, F4.

Group III (weak tolerance): four accessions (17.39%), including D2, D3, H4, ZK1.

The clustering result aligns well with the *D* value ranking, confirming the robustness of the evaluation system.

### 3.6. Stepwise Regression Identifies Key Screening Indicators

To identify a minimal set of indicators for rapid tolerance evaluation, stepwise regression was performed with the *D* value as the dependent variable and the seven relative trait values as independent variables. The resulting regression model was as follows:

Y = −0.227 + 1.227*X*_4_ + 0.326*X*_7_ + 0.142*X*_2_

where *X*_4_ = VI, *X*_7_ = FW, *X*_2_ = GE; adjusted *R*^2^ = 0.983, *p* < 0.001.

Variance inflation factors (VIFs) for all predictors were below 1.5, indicating no serious multicollinearity. The Durbin–Watson statistic was 1.92, suggesting that the residuals were independent. Given the limited sample size of 23 accessions, the regression results should be interpreted with appropriate caution; however, the high adjusted *R*^2^ and consistency with PCA results support the robustness of the screened evaluation indicators.

Among these, VI, FW, and GE entered the model first and explained the majority of variance, indicating they are the most informative and easily measurable indicators for distinguishing quinoa tolerance to compound saline–alkali stress.

## 4. Discussion

### 4.1. An Integrated Multivariate Framework Improves Evaluation Accuracy

Evaluating crop tolerance to compound saline–alkali stress using a single indicator is often one-sided and inconsistent [44,45]. The more indicators used, the more accurately stress resistance can be reflected. In this study, we established a structured analytical pipeline: PCA transformed seven correlated traits into three independent composite indices—seed germination capacity, seedling vigor and root development, and aboveground biomass accumulation—corresponding to PC1, PC2, and PC3, respectively; membership function analysis converted these components into a normalized D value; cluster analysis then objectively partitioned germplasm into tolerance groups. This workflow is similar to methods used for foxtail millet [46], melon [47], and wheat [48]. The cumulative contribution rate of 89.203% exceeds that reported in many single-salt studies (typically 70–80%), suggesting that multidimensional traits are particularly important for capturing complex stress responses.

### 4.2. Key Indicators Reflect Distinct Physiological Dimensions of Compound Stress

Stepwise regression identified VI, FW, and GE as the most pivotal indicators. Their physiological significance under compound saline–alkali stress warrants discussion.

The vigor index (VI = GI × BL) emerged as the most sensitive discriminator among all evaluated traits (CV = 0.460). Its exceptional sensitivity under compound saline–alkali stress can be attributed to its composite nature, which integrates two physiologically distinct processes: germination progression (GI) and early seedling elongation (BL). Under high-pH conditions (pH 10.0 in the 50 mmol/L treatment), alkaline stress imposes specific constraints on root and shoot meristems. The elevated pH damages root tip meristems, inhibits cell wall loosening by affecting expansin activity and xyloglucan endotransglucosylase/hydrolase (XTH) function, and disrupts auxin-mediated cell expansion [49]. These alkaline-specific effects are largely absent under neutral salt stress, where osmotic and ionic effects predominate [50]. Consequently, an accession that germinates well under compound stress but fails to sustain seedling elongation will have a dramatically reduced VI, whereas one that can both germinate and maintain growth will exhibit a high VI. This makes VI a particularly effective integrator of the combined impacts of salinity and alkalinity—a dual stress combination that cannot be adequately captured by either germination rate or growth parameters alone. Notably, the two elite salt–alkali-tolerant accessions identified in this study, LL1 and Z27, both ranked in the top 5 for relative VI values, further validating the strong discriminatory power of this indicator.

Fresh weight (FW) directly reflects net biomass accumulation, which under compound saline–alkali stress is constrained by multiple interacting factors. First, the high osmotic potential of the mixed salt solution (50 mmol/L, composed of four salts at a 1:1:1:1 molar ratio) restricts water uptake during imbibition and early seedling establishment [51]. Second, the alkaline pH (~10.0) disrupts plasma membrane integrity and H^+^-ATPase activity, impairing nutrient uptake and assimilation [32]. Third, the simultaneous presence of Na^+^, Cl^−^, SO_4_^2−^, HCO_3_^−^, and CO_3_^2−^ creates a complex ionic environment that interferes with K^+^ homeostasis—a critical determinant of cell expansion and osmotic adjustment [49]. Accessions that maintain higher FW under stress are those capable of sustaining water uptake, preserving membrane integrity, and maintaining metabolic activity despite the combined ionic and alkaline challenges [32]. The relatively high CV for FW (0.353) across the seven indicators confirms its value as an integrative endpoint of these interacting physiological processes.

Germination energy (GE), recorded on day 4, reflects the speed and uniformity of germination initiation—a critical parameter under compound saline–alkali stress. The physiological basis for GE’s importance lies in the timing of stress exposure: seeds that germinate rapidly minimize the duration of contact with toxic ions and high pH before radicle emergence [50]. Rapid reactivation of embryo metabolism, including the resumption of respiratory activity, mobilization of storage reserves, and activation of antioxidant defense systems, is essential to escape prolonged exposure to alkaline conditions that can cause irreversible damage to meristematic tissues [52]. Under the high-pH conditions of the present study, delayed germination substantially increases the risk of cell membrane degradation and ion toxicity, as the protective barriers provided by the seed coat become less effective once imbibition begins [53]. This temporal dimension of stress avoidance explains why GE, rather than the final germination rate (GR), emerged as a key indicator in the stepwise regression model. Similarly, both LL1 and Z27 also maintained top-level performance for relative GE and relative FW, which synergistically supported their top rankings in the comprehensive tolerance evaluation.

### 4.3. Comparison with Single-Salt Studies Reveals Stress-Specific Indicator Shifts

Unlike single-salt or single-alkali stress, compound saline–alkali stress is characterized by the simultaneous presence of neutral salts (NaCl, Na_2_SO_4_) and alkaline salts (NaHCO_3_, Na_2_CO_3_), which imposes a dual challenge on plants: ionic/osmotic stress from salinity and high-pH stress from alkalinity [54]. In natural soda saline–alkali soils such as those in the Songnen Plain of Northeast China, these two stress components invariably co-occur, and their combination produces effects that cannot be predicted from either stress alone [55]. Shi et al. demonstrated that, under mixed saline–alkali stress, synergistic effects between salt stress and alkali stress are a characteristic feature [56]. Therefore, evaluating germplasm tolerance using single-salt or single-alkali treatments may overlook the synergistic effects that operate under field conditions, underscoring the necessity of compound stress screening, as employed in the present study. Previous studies on quinoa germination under singular NaCl or NaHCO_3_ stress have reported different key indicators. Zhang Y. et al. [57] found that fresh weight, bud length, and germination rate were key under NaCl stress, while Wang Z. et al. [58] identified root length, germination energy, and fresh weight for alkali stress.

The differential indicator profiles between single-salt and compound stress treatments reflect fundamentally distinct stress physiology. Under compound saline–alkali stress, the synergistic interaction between high pH and ionic toxicity substantially amplifies the inhibitory effects on germination beyond what would be expected from an additive combination of the two stresses [56]. The synergistic effect is most evident in seedling growth parameters. At the same time, GR may be relatively maintained under moderate compound stress, consistent with our observation that there was no significant difference in GR between 50 mmol/L and the control, whereas the elongation of embryonic roots and buds was much more severely restricted [53]. This is because cell expansion and division are exquisitely sensitive to both osmotic imbalance and pH-induced cell wall stiffening. The extremely low mean relative values of RL (0.088) and BL (0.303) observed in our study (Table 2) are consistent with this interpretation and align with previous findings that saline–alkali concentration is strongly negatively correlated with BL and RL [54,59].

These differential indicator profiles have important implications for breeding programs. Screening under single NaCl stress may identify accessions that maintain germination but fail to establish seedlings under field saline–alkali conditions, whereas screening under compound stress, as employed here, more accurately reflects the multifaceted challenges of natural soda saline–alkali soils such as those in the Songnen Plain.

### 4.4. Tolerant Germplasm as Resources for Breeding

The strong tolerance of LL1 (from Gansu, China) and Z27 (from Peru) despite their distinct origins implies that tolerance mechanisms have evolved independently under different edaphic pressures. These two accessions should be prioritized for crossing programs and for further physiological dissection. The classification into three tolerance groups provides direct parental materials for breeding programs. Notably, thousand-grain weight varied widely among accessions (1.238~2.417 g) but showed no correlation with tolerance rankings, confirming that seed size does not confound our trait-based evaluation.

### 4.5. Limitations and Future Directions

Several limitations should be acknowledged to contextualize the findings and guide future research.

(1) This study was conducted under controlled laboratory conditions and cannot fully replicate the complexity of field saline–alkali environments, including heterogeneous salt distribution, variable soil properties, and fluctuating climatic conditions. We will launch multi-year field trials in native soda saline–alkali soils of the Jilin Songnen Plain to verify the full-growth-period agronomic performance of the eight elite tolerant accessions before their application in breeding programs.
(2) Our evaluation only covers the 0–8 days germination and early seedling stage, which is the most stress-sensitive window ideal for preliminary large-scale screening. However, saline–alkali tolerance at later vegetative, flowering, and grain filling stages may be controlled by distinct physiological and genetic pathways. A follow-up multi-stage evaluation is required to fully characterize the tolerance traits of these germplasm resources.
(3) Although we have identified VI, FW, and GE as core screening indicators and provided their physiological interpretations, the underlying regulatory networks remain uncharacterized. Subsequent physiological-biochemical assays and transcriptomic/proteomic comparisons between tolerant (LL1, Z27) and sensitive (D2, D3, H4) accessions will be carried out to clarify the molecular basis of differential tolerance to compound saline–alkali stress in quinoa.

## 5. Conclusions

(1) The tested quinoa germplasm materials generally exhibited saline–alkali tolerance, with marked variation among accessions.
(2) Based on comprehensive evaluation *D* values, the 23 quinoa accessions were classified into three categories: strong tolerance (8 accessions), moderate tolerance (11 accessions), and weak tolerance (4 accessions). The identified tolerant accessions (LL1 and Z27) are now being used in our crossing program. Future work will focus on the transcriptomic profiling of these two accessions to uncover novel salt-alkali tolerance genes.
(3) Vigor index (VI), fresh weight (FW), and germination energy (GE) can be used as reliable and easily measurable indicators for evaluating compound saline–alkali tolerance during germination.

## Figures and Tables

**Table 1:** Information on the 23 quinoa accessions.

Germplasm	Origin	TGW (g)	Germplasm	Origin	TGW (g)
16ND-3	Shanxi	2.253	K2	Bolivia	2.226
16ND-4	Shanxi	2.218	LL1	Gansu	2.417
A4	Bolivia	1.762	S1	Bolivia	2.206
C3	Bolivia	1.384	SL1	Bolivia	1.978
D2	Bolivia	2.155	Z1	Peru	2.344
D3	Bolivia	2.194	Z2	Peru	2.101
E5	Bolivia	1.751	Z4	Peru	2.154
F1	Bolivia	1.409	Z27	Peru	1.592
F2	Bolivia	1.238	ZK1	Peru	2.063
F4	Bolivia	1.529	ZK2	Peru	1.936
H2	Bolivia	2.233	ZK5	Peru	1.342
H4	Bolivia	1.529			

**Table 2:** Relative value of each index under compound saline–alkali stress.

Germplasm	*X*_1_ (GR)	*X*_2_ (GE)	*X*_3_ (GI)	*X*_4_ (VI)	*X*_5_ (RL)	*X*_6_ (BL)	*X*_7_ (FW)
16ND-3	0.944	0.898	0.858	0.143	0.136	0.206	0.667
16ND-4	0.917	0.893	0.785	0.098	0.100	0.201	0.138
A4	0.978	0.881	0.780	0.058	0.053	0.318	0.489
C3	0.976	0.835	0.540	0.080	0.084	0.394	0.653
D2	0.918	0.831	0.607	0.066	0.079	0.098	0.109
D3	0.880	0.626	0.336	0.040	0.045	0.182	0.418
E5	0.558	0.533	0.235	0.096	0.097	0.541	0.371
F1	1.039	0.896	0.545	0.065	0.067	0.368	0.324
F2	1.005	0.952	0.746	0.079	0.079	0.484	0.504
F4	1.000	1.012	0.701	0.048	0.050	0.397	0.455
H2	0.999	0.975	0.882	0.139	0.133	0.296	0.509
H4	0.584	0.321	0.210	0.065	0.073	0.216	0.365
K2	1.071	0.863	0.795	0.045	0.041	0.207	0.406
LL1	0.989	0.916	0.746	0.206	0.207	0.147	0.499
S1	0.767	0.676	0.461	0.081	0.085	0.210	0.460
SL1	0.988	0.915	0.641	0.077	0.089	0.214	0.409
Z1	0.907	0.779	0.408	0.064	0.074	0.261	0.502
Z2	0.819	0.699	0.400	0.058	0.058	0.390	0.514
Z4	0.798	0.585	0.300	0.062	0.076	0.420	0.609
Z27	0.930	0.893	0.763	0.133	0.158	0.479	0.579
ZK1	0.772	0.632	0.365	0.067	0.068	0.204	0.368
ZK2	0.976	0.952	0.887	0.070	0.072	0.322	0.655
ZK5	0.895	0.756	0.514	0.089	0.092	0.404	0.876
Min	0.558	0.321	0.210	0.040	0.041	0.098	0.109
Max	1.071	1.012	0.887	0.206	0.207	0.541	0.876
Mean	0.900	0.796	0.587	0.084	0.088	0.303	0.473
*CV*	0.147	0.213	0.367	0.460	0.443	0.398	0.353

Note: *X*_1_: relative GR, *X*_2_: relative GE, *X*_3_: relative GI, *X*_4_: relative VI, *X*_5_: relative RL, *X*_6_: relative BL, *X*_7_: relative FW.

**Table 3:** Principal component eigenvectors (component coefficients) and contribution rates for quinoa morphological parameters under compound saline–alkali stress.

Index	PC1	PC2	PC3
GE	0.256	−0.307	0.064
GR	0.287	−0.218	0.073
GI	0.293	−0.148	−0.012
VI	0.207	0.423	−0.147
RL	0.194	0.440	−0.148
BL	−0.026	0.101	0.606
FW	0.077	0.186	0.537
Eigenvalue	3.111	1.721	1.412
Contribution rate (%)	44.449	24.587	20.167
Cumulative contribution rate (%)	44.449	69.035	89.203

Note: PC1, PC2, and PC3 are designated as seed germination capacity factor, seedling vigor and root development factor, and aboveground biomass accumulation factor, respectively, based on the traits with the highest loading in each component.

**Table 4:** *D* value and ranking of compound saline–alkali tolerance of quinoa.

Germplasm	Comprehensive Index			Subordinate Function			*D* Value	Rank
	*CI*(1)	*CI*(2)	*CI*(3)	*μ*(1)	*μ*(2)	*μ*(3)		
16ND-3	2.284	1.198	−0.262	0.852	0.687	0.484	0.723	3
16ND-4	0.832	−0.606	−1.961	0.648	0.330	0.111	0.439	17
A4	0.437	−1.400	0.502	0.592	0.173	0.652	0.490	10
C3	0.302	0.001	1.335	0.573	0.450	0.835	0.599	8
D2	−0.250	−1.272	−2.467	0.496	0.198	0.000	0.302	21
D3	−1.973	−0.902	−0.621	0.253	0.271	0.405	0.293	22
E5	−2.782	2.233	0.626	0.139	0.891	0.680	0.469	13
F1	0.163	−1.276	0.134	0.554	0.197	0.571	0.460	14
F2	1.037	−0.678	1.393	0.677	0.316	0.848	0.616	6
F4	0.535	−1.769	0.989	0.606	0.100	0.759	0.501	9
H2	2.533	0.645	−0.227	0.887	0.577	0.492	0.713	4
H4	−3.772	1.365	−1.181	0.000	0.720	0.283	0.262	23
K2	0.484	−2.275	−0.321	0.599	0.000	0.471	0.405	19
LL1	3.334	2.784	−1.820	1.000	1.000	0.142	0.806	1
S1	−1.145	0.522	−0.708	0.370	0.553	0.386	0.424	18
SL1	0.713	−0.786	−0.640	0.631	0.294	0.401	0.486	11
Z1	−0.729	−0.322	0.021	0.428	0.386	0.547	0.443	15
Z2	−1.527	−0.075	0.845	0.316	0.435	0.728	0.442	16
Z4	−1.918	0.768	1.224	0.261	0.602	0.811	0.479	12
Z27	1.916	1.691	0.973	0.801	0.784	0.756	0.786	2
ZK1	−1.845	0.073	−0.969	0.271	0.464	0.329	0.337	20
ZK2	1.314	−0.908	1.049	0.716	0.270	0.772	0.606	7
ZK5	0.059	0.989	2.085	0.539	0.645	1.000	0.673	5
Max	3.334	2.784	2.085					
Min	−3.772	−2.275	−2.467					
Weight (%)				49.829	27.563	22.608		

*CI*(1), *CI*(2), *CI*(3): comprehensive index values for the three principal components. *μ*(1), *μ*(2), *μ*(3): member function values for the three components. *D*: comprehensive evaluation value.

## Data Availability

The original contributions presented in this study are included in the article/Supplementary Material. Further inquiries can be directed to the corresponding author.

## Supplementary Materials

The following supporting information can be downloaded at: https://www.mdpi.com/article/10.3390/plants15152413/s1. Table S1. The effect of different stress concentrations on germination indicators of quinoa germplasm.

## Abbreviations

The following abbreviations are used in this manuscript:

GR	Germination rate
GE	Germination energy
GI	Germination index
VI	Vigor index
RL	Root length
BL	Bud length
FW	Fresh weight
PCA	Principal component analysis
CV	Coefficient of variation
SA	Compound saline–alkali
TGW	Thousand-grain weight
