# Supplementary material for: Screening and Evaluation of Quinoa Germplasm for Saline–Alkali Tolerance Based on Germination Vigor Traits
Source: Plants (Basel). 2026 Aug 6;15(15):2413. doi: 10.3390/plants15152413 (PMC13468202; doi:10.3390/plants15152413)
Supplement: Supplementary file 1 [file plants-15-02413-s001.zip › plants-4417364-supplementary.pdf]

Table S1. The effect of different stress concentrations on germination indicators of quinoa germplasm

Treatment (mmol/L)	pH	GR	GE	GI	BL/ cm
CK	6.87	0.904 ± 0.013ab	0.904 ± 0.013a	26.011 ± 0.553a	2.731 ± 0.124a
SA-50	10.12	0.927 ± 0.018a	0.925 ± 0.019a	25.480 ± 0.737a	0.768 ± 0.069b
SA-100	10.05	0.922 ± 0.015ab	0.916 ± 0.016a	24.332 ± 0.684a	0.260 ± 0.026c
SA-200	9.94	0.819 ± 0.028bc	0.730 ± 0.035b	15.472 ± 1.638b	0.161± 0.016c
SA-300	9.84	0.632 ± 0.078d	0.527 ± 0.083c	8.882 ± 1.481c	0.016 ± 0.009c

Note: Different letters mean significant difference at the 5 % level.
